# Commencement Exercises

**Published:** 1857-10

**Authors:** 


### EDITORIAL AND MISCELLANEOUS.

### Commencement Exercises.

The Course of Lectures in the Atlanta Medical College, closed on the 3d of September, with an appropriate and highly interesting valedictory address to the Graduating Class, by Thos. S. Powell, M. D., of Sparta, Georgia.

The degree of M. D., was conferred upon forty-six gentlemen, the majority of whom we are sure, will in their career, reflect credit upon the Institution, let their lot be cast where it may.

